# The complete plastid genome of *Thrixspermum tsii* (Orchidaceae, Aeridinae)

**DOI:** 10.1080/23802359.2019.1703594

**Published:** 2020-01-21

**Authors:** Bao-Jian Ye, Sai Zhang, Xiong-De Tu, Ding-Kun Liu, Ming-He Li

**Affiliations:** a Fujian Colleges and Universities Engineering Research Institute of Conservation and Utilization of Natural Bioresources, College of Forestry, Fujian Agriculture and Forestry University, Fuzhou, China;; b Key Laboratory of National Forestry and Grassland Administration for Orchid Conservation and Utilization at College of Landscape Architecture, Fujian Agriculture and Forestry University, Fuzhou, China

**Keywords:** Vandeae, *Thrixspermum*, chloroplast genome, phylogeny

## Abstract

The complete plastid genome of *Thrixspermum tsii* was determined and analyzed in this work. The plastome was 149,689 bp in length with 86,778 bp of the large single-copy (LSC) region, 12,129 bp of the small single-copy (SSC) region and 25,391 bp of the inverted repeat (IR) regions. The genome contained 120 genes, 74 protein-coding genes, 38 tRNA genes, and 8 rRNA genes. Phylogenetic analysis of 17 Aeridinae plastomes suggested four groups were divided, and *T. tsii* was sister to *T. japonicum*.

*Thrixspermum* includes approximately 170 species distributed in the subtropical and tropical regions from Asia to Australia (Pridgeon et al. 2014). The exact position and relationships of *Thrixspermum* have remained unresolved using traditional sequences from one or a few markers. Members of *Thrixspermum* are similar to *Phalaenopsis* with a column foot and a three-lobed labellum. *Thrixspermum tsii* is an endemic species of south-central China. The species is similar to *T. centipeda* and *T. subulaturn* by having unequally obtuse-bilobed limbs at the top of leaves in vegetative characters and reproductive characters (Chen and Shui 2005).

Fresh leaf sample of *T. tsii* was acquired from Jinchang community, Malipo County, Yunnan Province of China (23°07′N, 104°42′E). The voucher specimen deposited at Fujian Agriculture and Forestry University (specimen code MH Li or079). DNA extraction, library constructing, sequencing and data filtering were referenced in Liu et al. (2019). The plastid genome of *T. japonicum* (KX871234) as reference, the paired-end reads were filtered with GetOrganelle pipe-line (Jin et al. 2018) to get plastid-like reads, then the filtered reads were assembled by SPAdes version 3.10 (Bankevich et al. 2012), the final ‘fastg’ were filtered by the script of GetOrganelle to get pure plastid contigs, and the filtered De Brujin graphs were viewed and edited by Bandage (Wick et al. 2015). Assembled plastid genome annotation based on comparison with *T. japonicum* by GENEIOUS v11.1.5 (Biomatters Ltd., Auckland, New Zealand) (Kearse et al. 2012). The matrix of 17 representative species of Aeridinae and 3 outgroup species (*Calanthe triplicata*, *C. davidii*, and *Cattleya crispata*) were aligned using MAFFT v7.307 (Katoh and Standley 2013). The phylogenetic tree was constructed by the maximum likelihood software IQ-TREE (Nguyen et al. 2015) based on the complete plastid genomes, and branch supports with the ultrafast bootstrap (Hoang et al. 2018).

The complete plastid genome sequence of *T. tsii* (GenBank accession number MN725094) was 149,689 bp in length, with a large single-copy (LSC) region of 86,778 bp, a small single-copy (SSC) region of 12,129 bp, and a pair of inverted repeat (IR) regions of 25,391 bp. The complete genome GC content was 36.2% (LSC, 33.4%; SSC, 27.0%; IR, 43.2%) and the plastome contained 120 genes, 74 protein-coding genes, 38 tRNA genes, and 8 rRNA genes.

The phylogenetic analysis of 17 Aeridinae plastomes showed that the *T. tsii* was sister to *T. japonicum* and four groups were divided with full support (Figure 1). Clade I contained four species of *Phalaenopsis*. Clade II comprised two species of *Thrixspermum*. Clade III contained two species of *Gastrochilus* and *Pelatantheria scolopendrifolia*. Clade IV contained two species of *Vanda* and six species of *Holcoglossum*.

**Figure 1.**
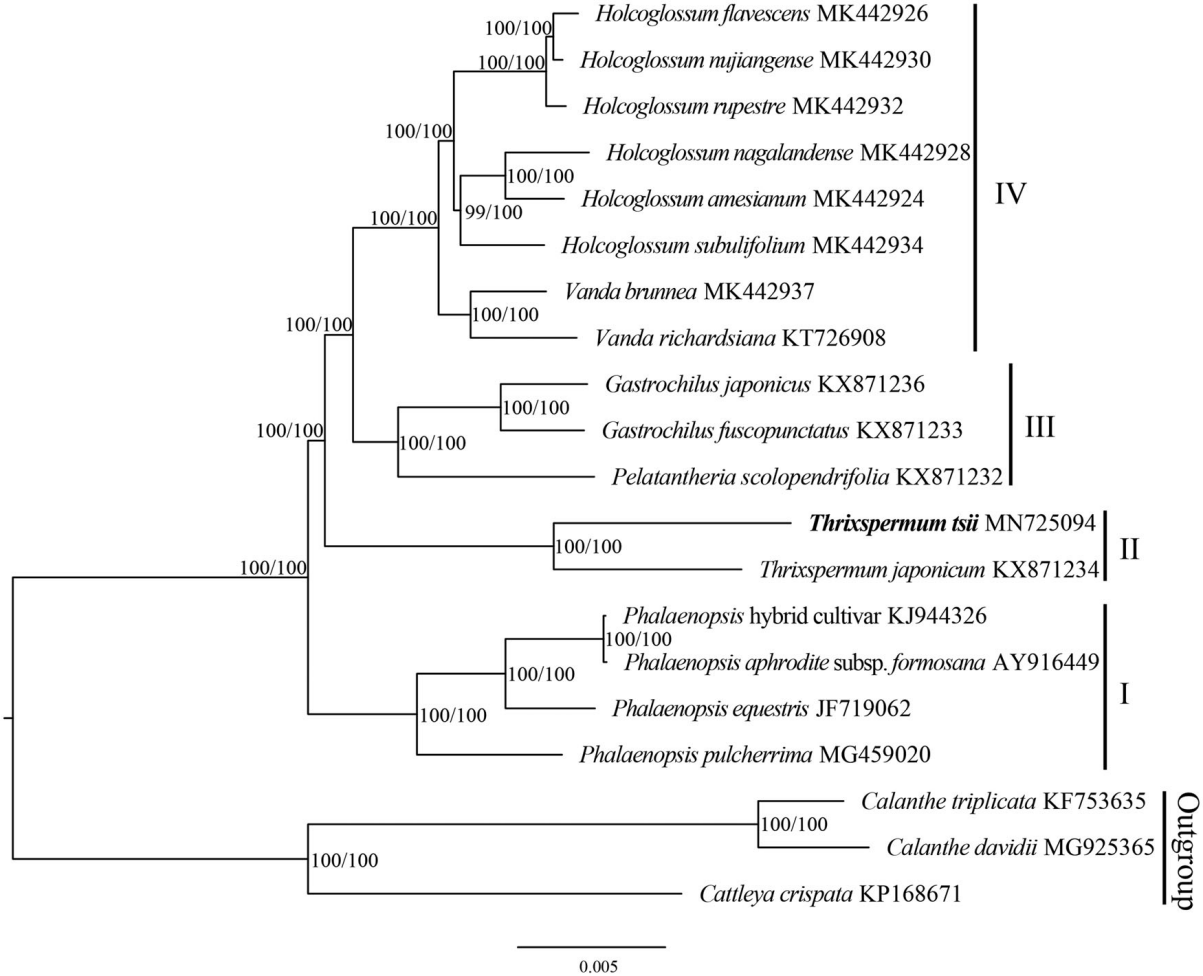
The maximum-likelihood (ML) tree based on the plastid genomes of 17 Aeridinae species and 3 outgroup species. Numbers near the nodes mean bootstrap support value (standard bootstrap left and ultrafast bootstrap right).
